# Construction of an SNP-based high-density linkage map for flax (*Linum usitatissimum* L.) using specific length amplified fragment sequencing (SLAF-seq) technology

**DOI:** 10.1371/journal.pone.0189785

**Published:** 2017-12-21

**Authors:** Liuxi Yi, Fengyun Gao, Bateer Siqin, Yu Zhou, Qiang Li, Xiaoqing Zhao, Xiaoyun Jia, Hui Zhang

**Affiliations:** 1 Biotechnology Research Center, Inner Mongolia Academy of Agricultural and Husbandry Sciences, Hohhot, Inner Mongolia, China; 2 Special Crops Institute, Inner Mongolia Academy of Agricultural and Husbandry Sciences, Hohhot, Inner Mongolia, China; 3 Corn Institute, Inner Mongolia Academy of Agricultural and Husbandry Sciences, Hohhot, Inner Mongolia, China; Mahatma Phule Krishi Vidyapeeth College of Agriculture, INDIA

## Abstract

Flax is an important crop for oil and fiber, however, no high-density genetic maps have been reported for this species. Specific length amplified fragment sequencing (SLAF-seq) is a high-resolution strategy for large scale *de novo* discovery and genotyping of single nucleotide polymorphisms. In this study, SLAF-seq was employed to develop SNP markers in an F_2_ population to construct a high-density genetic map for flax. In total, 196.29 million paired-end reads were obtained. The average sequencing depth was 25.08 in male parent, 32.17 in the female parent, and 9.64 in each F_2_ progeny. In total, 389,288 polymorphic SLAFs were detected, from which 260,380 polymorphic SNPs were developed. After filtering, 4,638 SNPs were found suitable for genetic map construction. The final genetic map included 4,145 SNP markers on 15 linkage groups and was 2,632.94 cM in length, with an average distance of 0.64 cM between adjacent markers. To our knowledge, this map is the densest SNP-based genetic map for flax. The SNP markers and genetic map reported in here will serve as a foundation for the fine mapping of quantitative trait loci (QTLs), map-based gene cloning and marker assisted selection (MAS) for flax.

## Introduction

Flax (*Linum usitatissimum* L), which belongs to the Linaceae family, is an annual, self-pollinating, and diploid (2n = 2x = 30) crop with a genome size of ~ 370 Mb [1, 2]. Flax has been used by human beings for several thousands years as a source of high quality oil (linseed) and stem fiber (linen) [3]. Flaxseed oil is well known for its nutraceutical functions because it contains high percentage of unsaturated fatty acids and lignans. The unsaturated fatty acids in flaxseed oil, especially, the linolenic acid (LIN, ~55%), also known as α- linolenic acid, is the precursor of the long chain polyunsaturated fatty acids eicosapentaenoic acid (EPA), docosapentaenoic acid (DPA) and docosahexaenoic acid (DHA) which are synthesized in the human body and recognized for their beneficial effects on cardiovascular health [4]. Flax seed is also rich in lignans such as secoisolariciresinol diglucoside (SDG), which is an antioxidant and the precursor of several phytoestrogens [5], and consumption of flax seed has the effects on the treatment of certain cancers and inflammatory diseases because of high content of lignans[6]. In addition, because of its feature of oxidative instability, linseed oil is valuable in industry for paints, linoleum flooring, inks and varnishes [7].

High-density genetic maps are constructed for application in quantitative trait loci (QTLs) mapping, map-based gene cloning and marker-assisted selection (MAS). Several genetic linkage maps for flax have been reported to date [8–12]. The main molecular markers used in these genetic maps included amplified fragment length polymorphism (AFLP), restriction fragment length polymorphism (RFLP), random amplified polymorphic DNA (RAPD), sequence tagged site (STS), simple sequence repeat (SSR). RAPD and AFLP markers lack repeatability, stability and have no sequence information. SSR and AFLP markers stable and reliable for map construction, but they are laborious and costly[13]. Furthermore, these maps were constructed based on a limited number of markers, resulting in a relatively low-density genetic maps. It is imperative to build a high-density genetic map for flax using reliable and abundant type of markers. Single nucleotide polymorphisms (SNPs) as the most abundant type of DNA variations are currently used in genetic map construction because of their wide and even distribution in the genome [14]. It’s been made possible to detect a large number of SNPs throughout the genome for high-density genetic maps construction due to the advances in next generation sequencing technologies and high-throughput genotyping methods. Technologies that can develop large scale SNP markers in a short time include complexity reduction of polymorphic sequences (CroPS) [15], restriction-site-associated DNA sequencing (RAD-seq) [16, 17], genotyping-by-sequencing (GBS) [18], and specific length amplified fragment sequencing (SLAF-seq) [19]. SLAF-seq is distinguished in high-throughput, high accuracy, while low cost and time-saving. Development of SNP markers and construction of high-density genetic map based on SLAF-seq have been successfully applied in a number of crops, such as sesame [20], soybean [21], mango [22], tea [23], grape [24], willow [25], and cucumber [26, 27].

In this study, SLAF-seq was applied for SNP discovery and high density genetic map construction for flax, using an F_2_ population derived from the cross between LH89 and R43. The new high-density genetic map can be used in map-based QTL mapping of important traits and marker-assisted breeding in flax.

## Materials and methods

### Plant materials and genomic DNA extraction

A mapping population of 100 individuals which were randomly selected from an F_2_ segregation progeny derived from a cross between LH98 (the male parent, high linoleic acid, blue petals) and R43 (the female parent, high linolenic acid, white petals) was used in the genetic map construction. The F_2_ population, together with their parents, was grown in the experimental field at the Inner Mongolia Academy of Agricultural and Husbandry Sciences in Hohhot, China. Young leaves were collected from two parents and F_2_ individuals, frozen in liquid nitrogen. Genomic DNA was extracted using CTAB method [28]. The extracted DNA was tested by electrophoresis in 1% agarose gel and analyzed on the ND-1000 spectrophotometer platform (NanoDrop, Wilmington, DE, USA) for concentration and purity.

### SLAF library construction and high-throughput sequencing

SLAF library construction was conducted as described by Sun et al [19] with some improvements. Firstly, reference genome of flax (*Linum usitatissimum*. L) [2] (http://phytozome.jgi.doe.gov/pz/portal.html#!info?alias=Org_Lusitatissimum) was used to design marker discovery experiments by simulating *in silico* the number of markers produced by different enzymes. Next, a SLAF pilot experiment was performed, and the SLAF library conducted in accordance using the predesigned scheme. For the F_2_ population, two enzymes *Hae*Ⅲ + *Rsa*Ⅰ (New England Biolabs, NEB, USA) were used to digest the genomic DNA. A single nucleotide (A) overhang was added subsequently to the digested fragments using Klenow Fragment (3' → 5' exoˉ) (NEB) and dATP at 37°C. Duplex tag-labeled sequencing adapters (PAGE-purified, Life Technologies, USA) were then ligated to the A-tailed fragments using T4 DNA ligase. Polymerase chain reaction (PCR) was performed using diluted restricton-ligation DNA samples, dNTP, Q5®High-Fidelity DNA Polymerase and PCR primers (Forward primer: 5'-AATGATACGGCGACCACCGA-3', reverse primer: 5'-CAAGCAGAAGACGGCATACG-3') (PAGE-purified, Life Technologies, USA). PCR products were then purified using Agencourt AMpure XP beads (Beckman Coulter, High Wycombe, UK) and pooled. Pooled samples were separated by 2% agarose gel electrophoresis. Fragments ranging from 314–414 base pairs (with indexes and adaptors) in size were excised and purified using a QIAquick gel extraction kit (Qiagen, Hilden, Germany). Gel-purified products were then diluted. After the SLAF library construction, high-throughput paired end sequencing was carried out on Illumina-HiSeq^TM^ 2500 sequencing platform (Illumina, Inc; San Diago, CA, U.S.) at Biomarker Technologies Corporation in Beijing (http://www.biomarker.com.cn/english). To evaluate accuracy of SLAF library construction, the sequence error rate was estimated using the data from rice (*Oryza sativa indica*) [29] as control (http://rice.plantbiology.msu.edu/), of which genome size is 382Mb. The ratio of high quality reads with quality scores greater than Q30 (means a quality score of 30, indicating a 1% chance of an error, and thus 99% confidence) in raw reads and guanine- cytosine (GC) content were calculated for quality control.

### SLAF-seq reads grouping

All SLAF pair-end reads with clear index information were clustered based on sequence similarity as detected by BLAT (BLAST-like alignment tool) [30] (tileSize = 10, stepSize = 5). Sequences with over 90% identity were grouped in one SLAF locus as described by Sun et al [19]. Alleles were defined in each SLAF using the minor allele frequency (MAF) evaluation. Because flax is a diploid species, one locus contains at most four SLAF tags, so groups containing more than four tags were filtered out as repetitive SLAFs. In this study, SLAFs with a sequence depth of less than 9-fold were considered as low-depth SLAFs and filtered out. SLAFs with 2, 3, or 4 tags were identified as polymorphic SLAFs and considered to be potential markers.

### SNP calling and genotype definition

Low quality reads (quality scores < 30) were filtered out and the SNPs were called using Burrows–Wheeler Aligner (BWA) [31], Genome Analysis Tookit (GATK) [32] and Sequence Alignment/Map tools (SAMtools) [33]. The F_2_ individual sequence reads were aligned against the flax genome [2] using BWA with parameters defined as Score (missed match) = 3, Score (opening gap) = 11 and Score (Gap extension) = 4. False alignment was always detected near InDels, therefore, local alignment was performed. The “Unified Genotype” function of GATK was used for variant calling. Any nucleotide difference between reads and the reference genome was identified as a variant. SMAtools and GATK were used to identify SNP, and their intersection was merged as the candidate SNP dataset. Only biallelic SNPs were retained as the final SNP dataset. Polymorphic markers were classified into eight segregation patterns (ab × cd, ef × eg, hk × hk, Im × II, nn × np, aa × bb, ab × cc, and cc × ab). An F_2_ population is obtained by selfing the F_1_ of a cross between two fully homozygous parents with genotype aa or bb. Therefor, only the SNPs whose segregation pattern was aa × bb were used for genetic map construction. The average sequence depths of SNPs were greater than 20-fold in parents and greater than 9-fold in progeny. And a progeny contained more than 80% of the SNPs in the parents, ie, 80% integrity of SNPs in individuals. A chi square test was conducted for each SNP with a null hypothesis that the two alleles at a locus segregated with a ratio of 1:1 in the F_2_ population. All SNPs that significantly deviated from this ratio (p < 0.001) were excluded from the SNP data.

### Linkage map construction

In order to ensure the quality of the genetic map, we filtered SNPs according to the following four criteria: 1) SNPs that were heterozygous in the parents; 2) SNPs with sequence depths of less than 4-fold in F2 population; 3) SNPs showing extremely significant (p < 0.001) segregation distortion; 4) SNPs showing less than 70% integrity of individual segregation patterns. To ensure efficient construction of the high-density and high-quality map, a newly developed HighMap [34] strategy was utilized to order the SNP markers and correct genotyping errors within LGs. Firstly, recombinant frequencies and MLOD [35] scores were calculated by two-point analysis, which were applied to infer linkage phases. Then, enhanced gibbs sampling, spatial sampling and simulated annealing algorithms were combined to conduct an iterative process of marker ordering [36, 37]. Briefly, in the stage of the ordering procedure, SNP markers were selected using spatial sampling. One marker was taken randomly in a priority order of test cross, and markers with a recombination frequency smaller than a given sampling radius are excluded from the marker set. Subsequently, simulated annealing was applied to searching for the best map order. Summation of adjacent recombination fractions was calculated [34]. The annealing system continued until, in a number of successive steps, the newly generated map order is rejected. Blocked Gibbs sampling was employed to estimate multipoint recombination frequencies of the parents after the optimal map order of sample markers were obtained [34]. The updated recommbination frequencies were used to integrate the two parental maps, which optimize the map order in the next cycle of simulated annealing. Once a stable map order was obtained after 3–4 cycles, we turned to the next map building round. A subset of currently unmapped markers was selected and added to the previous sampling with decreased sample radius. The mapping algorithm repeats until all the markers were mapped appropriately. The error correction strategy of SMOOTH [38] was then conducted according to parental contribution of genotypes, and a k-nearest neighbor algorithm [39] was applied to impute missing genotypes. Skewed markers were then added into this map by applying a multipoint method of maximum likelihood. Map distances were estimated using the kosambi mapping function [40]. The haplotype maps and heat maps were used to evaluate the genetic map [41]. “Draw_haplotype-map.pl” and “draw_heatmap.pl” were used to construct haplotype maps and heat maps, respectively. Haplotype maps were generated for each of the 100 F_2_ haploid individuals and were used to reflect possible double crossover, suggesting genotyping errors. Heat maps were generated to reflect the recombination relationships between markers in each of the LGs and used to detect the potential ordering errors.

## Results

### SLAF library construction and SLAF tag development

**Fig 1:**
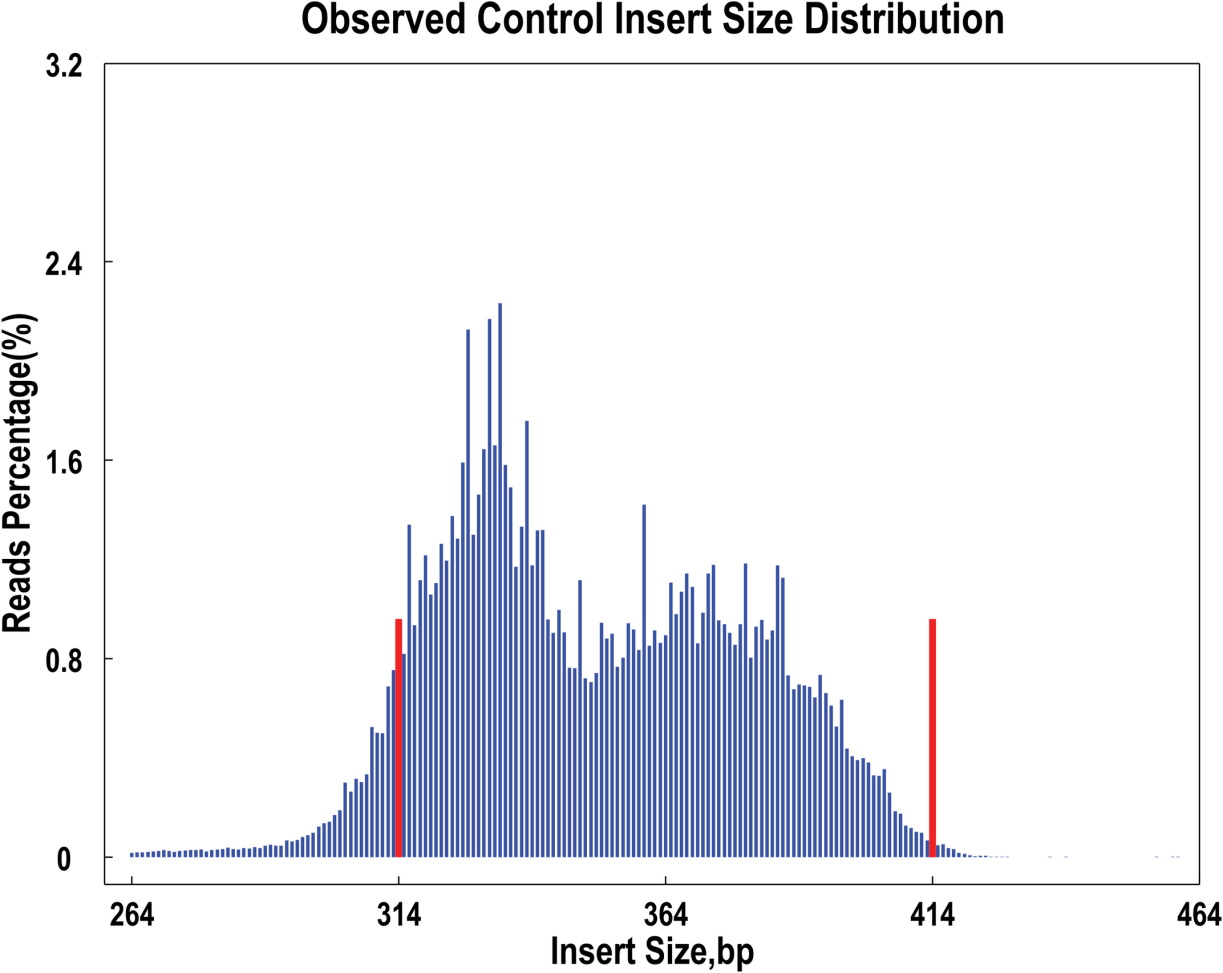
The observed insert size distribution. **Note:** X-axis represents the size of the insert reads; Y-axis represents the percentage of insert reads of each size. The expected size of SLAFs fall within the interval range between the two red lines. It can be seen in the figure that size of the most sequence reads were between 314-414bp.

After *in silico* double digestion of flax reference genome with *Hea*Ⅲ + *Rsa*Ⅰ, restriction fragments of 314–414 bp were defined as SLAF tags, and 118,778 of them were expected. SLAF tags were evenly distributed on the genome and repetitive SLAF percentage was 4.27%. evaluation of the rice SLAF showed that cleavage efficiency of the *Hea*Ⅲ + *Rsa*Ⅰ restriction enzymes was 92.1%, and the paired-end reads accounted for 96.02% of all reads generated (Fig 1), indirectly proving that the SLAF library constructed for flax was of high quality and suitable to high-throughput sequencing.

After SLAF library construction and high-throughput sequencing, 196.29 million paired-end reads were obtained, with each reads being 125 bp × 2 in length. The average Q30 ratio was 82.22% and the average guanine-cytosine (GC) content was 38.92%. Of these high quality data, 9,348,755 were from the male parent and 11,017,232 were from the female parent. The average read number for the 100 individuals in F_2_ population was 1,179,274. For the control rice, the read number, Q30, and GC content were 1,179,484, 81.64% and 41.43%, respectively (Table 1).

**Table 1:** Basic statistic of the SLAF-seq data in flax.

Sample ID	Total reads	Q30 percentage (%)	GC percentage (%)
**LH-89 (male parent)**	9,348,755	82.45	39.53
**R43 ((femal parent)**	11,017,232	82.29	38.60
**Average of 100 F**_**2**_ **population**	1,759,274	81.94	38.63
**Rice (control)**	1,179,484	81.64	41.43

**Note:** total reads means the number of total reads; Q30 means the percentage of bases with sequencing values ≦ 30 in the total bases; GC percentage means the percentage of G and C bases in the total bases; Rice data was used to assess the quality of SLAF library construction.

By bioinformatics analysis, a total of 389,288 SLAFs were obtained. The numbers of SLAFs in the male and female parents were 261,699 and 229,601, respectively. The total depths were 7,386,264 and 6,562,659 in the male and female parents, respectively. The average depth for each SLAF was 32.17-fold in the male parent and 25.08-fold in the female parent. In the F_2_ population, the average number of SLAF, average total depth and average depth were 126,193, 1,225,623 and 9.64-fold, respectively (Table 2).

**Table 2:** Summary of sequencing depth of SLAF markers.

Sample ID	SLAF number	Total Depth	Average Depth
**LH-89 (male parent)**	261,669	6,562,659	25.08
**R43 (female parent)**	229,601	7,386,264	32.17
**Average of 100 F**_**2**_ **individuals**	126,193	1,225,623	9.64

**Note:** SLAF number represents the number of total SLAF tags; total depth represents the number of sequence reads; average depth represents the average number of sequence reads of the sample for the SLAF.

### SNP marker detection

**Fig 2:**
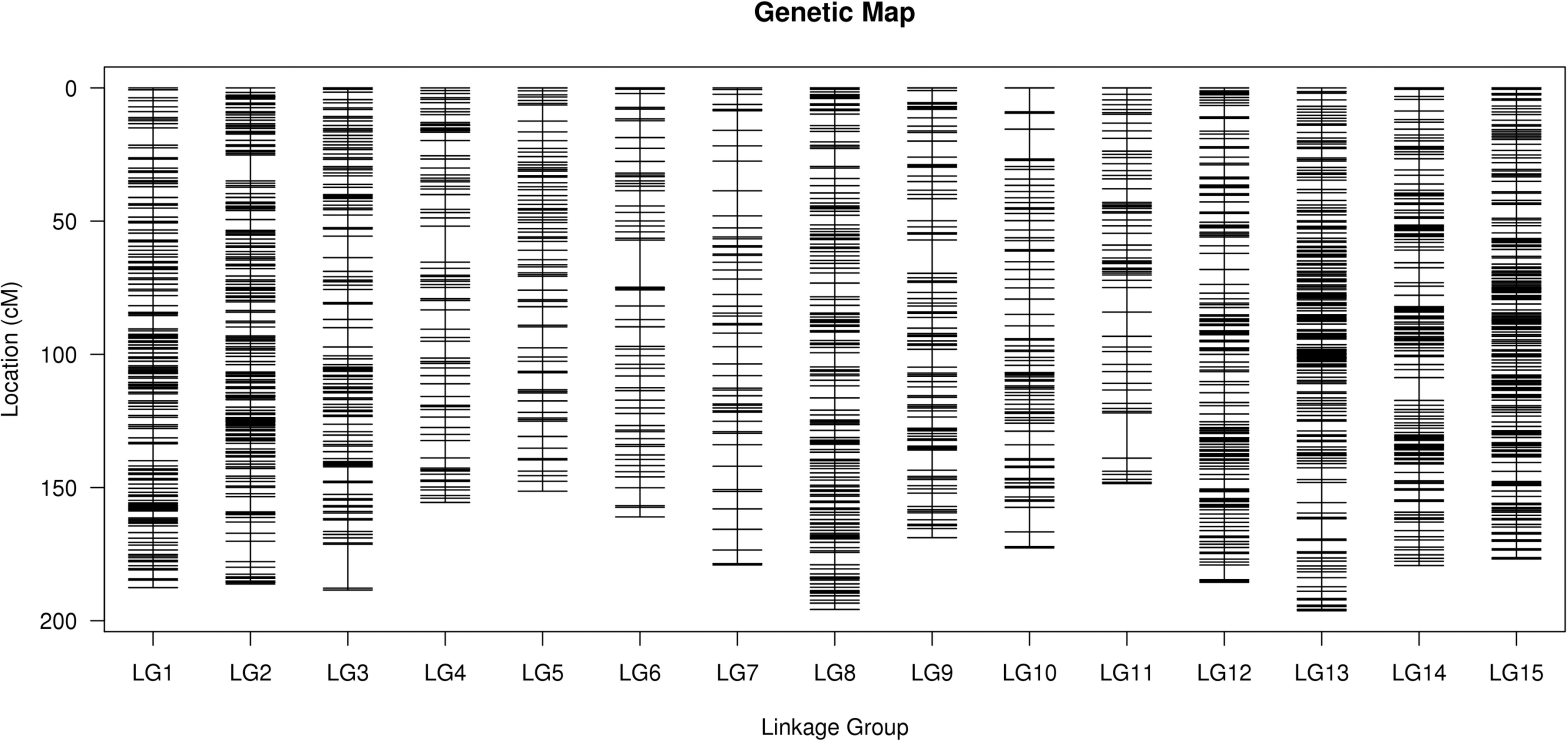
High-density genetic linkage map of flax. **Note:** A black bar indicates a SNP marker. The x-axis represents the linkage group number and the y-axis indicates the genetic distance (cM) in each linkage group. SNP markers and their locations are shown on the right and left side, respectively.

The numbers of SNP in the male and female parents were 199,356 and 187,019, respectively. In the F_2_ population, the average number of SNP in each individual was 117,836. After filtering out the SNPs with sequence depths less than 4-fold, a total of 65,845 markers were successfully coded from 260,380 polymorphic SNPs and classified into four segregation patterns: hk × hk, lm × ll, nn × np and aa × bb. An F_2_ population is obtained by selfing the F_1_ of a cross between two fully homozygous parents with genotype aa or bb. Therefore, only the SNPs whose segregation patterns were aa × bb were used for genetic map construction, which consisted of 22,441 SNP markers corresponding to a polymorphism rate of 8.62% (Tables 3 and 4; Fig 2).

**Table 3:** Information on sample SNP detected.

Sample ID	Total SNP	SNP number	Heter ratio
**LH-89 (male parent)**	260,380	199,356	22.45%
**R43 (female parent)**	260,380	187,019	17.90%
**Average of 100 F**_**2**_ **individuals**	260,380	117,836	18.68%

**Note:** total SNP means the total number of SNP detected; SNP number means the total number of SNP detected in the sample; heter ratio means sample heterozygosity.

**Table 4:** Genotype distribution of SNP markers.

Type	Number
**hk × hk**	13,873
**lm × ll**	11,469
**nn × np**	18,062
**aa × bb**	22,441
**total**	65.845

### Linkage map

After a series of filtering of 22,441 SNPs, a total of 4,638 SNPs were found suitable to build the genetic map. The MLOD [35] values between the 4,638 SNPs were calculated. Finally, 4,145 SNPs were allowed into 15 linkage groups (LGs). The linear arrangements of all the SNPs and genetic distances of adjacent SNPs within each LG were analyzed using HighMap software [34]. An integrated genetic map, 2,632.94 cM in total length and 0.64 cM in average length, was finally constructed. The number of SNP markers in different LGs ranged from 81 to 530 on the map. The longest LG was LG13 with 525 SNPs, a length of 196.24 cM, and an average distance of 0.37 cM between adjacent markers. The shortest LG was LG5, which was 148.52 cM in length, with only 85 SNPs, and an average marker distance of 1.77 cM. Linkage groups LG2, LG13 and LG15 were the most density groups, which had average marker density of 0.35 cM, 0.37 cM and 0.36 cM, respectively. The largest gap on this map was 17.69 cM located in LG6 (Table 5; Fig 2 and S1 Fig).

**Table 5:** Basic information of the flax genetic map based on SNP markers.

Linkage Group ID	Total Marker	Total distance (cM)	Average distance (cM)	Max Gap
**LG1**	432	187.55	0.44	6.49
**LG2**	530	186.22	0.35	9.72
**LG3**	252	188.46	0.75	16.47
**LG4**	136	155.60	1.15	13.52
**LG5**	138	151.37	1.10	7.87
**LG6**	103	161.02	1.58	17.69
**LG7**	81	179.07	2.24	11.14
**LG8**	402	195.77	0.49	6.74
**LG9**	178	168.80	0.95	12.49
**LG10**	162	172.67	1.07	11.18
**LG11**	85	148.52	1.77	16.96
**LG12**	317	185.59	0.59	6.03
**LG13**	525	196.24	0.37	7.64
**LG14**	314	179.28	0.57	8.56
**LG15**	490	176.77	0.36	5.21
**Total**	4,145	2,632.94	0.64	17.69

### Evaluation of the genetic map

The genetic map of flax was evaluated using the haplotype maps and heat map. Haplotype maps were created for each of the 100 F_2_ individuals using 4,145 SNP markers. The recombination events of each individual LG of the genetic map were displayed on the haplotype maps. The majority of recombinant blocks were clearly defined (S2 Fig). The missing percentage of the markers in each LG of this genetic map ranged from 2.8% to 10.62%, which did not significantly affect the quality of the genetic map (Table 6). It can also be seen that all the LGs distributed uniformally. Heat maps were also generated to evaluate the quality of the genetic map using pair-end recombination values for the 4,145 mapped SNP markers. Heat maps could reflect the recombination relationships between markers in each of the LGs and were used to find any potential ordering errors. It can be seen that most of the LGs performed well in visualization, indicting that the markers were well-ordered in each LG (S3 Fig). Consequently, the genetic map of flax was of high quality.

**Table 6:** Statistics of missing percentage of each LG.

Linkage group ID	Missing precentage (%)	Linkage group ID	Missing precentage (%)	Linkage group ID	Missing precentage (%)
**LG1**	5.64%	**LG6**	10.62	**LG11**	6.72
**LG2**	5.66	**LG7**	8.26	**LG12**	3.91
**LG3**	7.17	**LG8**	4.82	**LG13**	2.80
**LG4**	8.83	**LG9**	6.75	**LG14**	4.64
**LG5**	7.00	**LG10**	8.92	**LG15**	4.45

## Discussion

Genetic maps are essential for fine mapping of quantitative trait locus (QTL), map-based gene cloning and marker-assisted selection (MAS), however, the quality of genetic maps depends on the types and number of markers used. During the past two decades, genetic maps constructed for flax have been based on various types of molecular markers including RAPD, RFLP, AFLP and SSR [8–12]. The number of markers on the genetic maps for flax was only several hundreds, which has hindered the effective utilization. It will be a time-consuming and costly to generate a high-density genetic map for flax, using conventional low-throughput molecular marker technologies.

Single nucleotide polymorphisms (SNPs) are the most suitable and favorable marker for constructing genetic maps because of their abundance and even distribution across the genome. Rapid development in high-throughput sequencing technologies and genotyping methods, such as restriction site-associated sequencing (RAD-seq) [16, 17], genotyping by sequencing (GBS) [18]. Specific length amplified fragment sequencing (SLAF-seq) [19] has made it possible to detect large number of SNP markers for linkage map construction in short period of time with low cost. It is a reduced representative library (RRL) sequencing technology that digests genomic DNA with double restriction enzymes followed by sequencing of the paired-end of specific length fragments by next generation sequencing (NGS) technologies. SLAF-seq has been used to construct the genetic maps of a number of species in the last few years. Zhang, et al. [20] using this method to construct the first sesame high-density SNP genetic linkage map. Qi, et al. [21] constructed a high-density genetic map for soybean. Luo, et al. [22] constructed the high-density genetic map for mango based on large scale marker development. Ma, et al. [23] made a research in large-scale SNP discovery and constructed a high-density genetic map for tea plant. Guo, et al. [24] developed a high-density genetic map for vitis. Zhang, et al. [25] built a genetic linkage map for Tetraploid *Salix matsudana* using SLAF-seq. Xu, et al. [26] and Zhu, et al. [27] respectively constructed high-density SNP genetic maps for cucumber.

This paper has also adopted the improved SLAF-seq technology to develop SNP markers and construct the high-density genetic map for flax. In this research, we developed thousands of SNP markers for flax. We obtained a total of 196.29 million reads based on high-throughput sequencing, which produced 389,288 high-quality polymorphic SLAFs. From these SLAFs, a total of 4,145 polymorphic SNP markers were detected and used to construct the high-density genetic map. The quality and quantity of markers met the requirements for construction of a high-density genetic linkage map. Therefore, the technique of SLAF-seq is suitable for discovering large scale SNP markers in flax at low cost.

During the past two decades, several genetic maps for flax have been developed. The linkage map for flax developed by Spielmeyer et al. (1998) [8] using a mapping population of 59 double-haploid (DH) lines was composed of 213 AFLP markers which were grouped into 18 linkage groups. This map spanned 1,400 cM, with an average distance of 10 cM between markers. The genetic map reported by Oh et al. (2000) [9], using F_2_ populations derived from two crosses was based on 94 markers (13 RFLPs, 80 RAPDs and 1 STS) and grouped into 15 linkage groups, spanning about 1000 cM. The likage map developed by Cloutier et al. (2011) [10] using a DH mapping population of 78 individuals had 113 markers, mostly SSRs (103 SSRs, 5 SNPs, 4 genes and 1 seed coat color trait). That map was composed of 24 linkage groups, and spanned 833.8 cM. The average marker density was estimated at one marker per 7.3 cM in that map. The first consensus genetic map of flax (Cloutier S et al. 2012) [11] was constructed by merging data from three different mapping populations. That map contained 770 SSR markers which were grouped into 15 linkage groups, and spanned 1,551 cM with a mean marker density of 2.0 cM. Wu, et al. [12] constructed a genetic map of 12 linkage groups for flax using a population with 30 F_2_ individuals from a cross between Diane (cultivar for fibre) and Ningya 17 (cultivar for oil) with 71 SRAP and SSR markers. The map spanned a total length of 546.5 cM, with an average distance of 5.75 cM between adjacent markers. The genetic map in this study included 4,145 SNP markers on 15 linkage groups and was 2,632.94 cM in length, with an average distance of 0.64 cM between adjacent markers. Among all the genetic maps constructed for flax, the one reported here contained the most number of markers (4,145 SNPs), spanned the longest distance (2,632.94 cM) with the shortest average distance between adjacent markers (0.64 cM), thus the highest density genetic map ever developed for flax to date.

In conclusion, the present study proved that SLAF-seq technology can be successfully used for large number of SNP discovery and genotyping in flax. The genetic map generated in this report is the first SNP-based genetic map for flax, as well as the most saturated one developed to date. The SNP markers and genetic map reported in here will serve as a foundation for the fine mapping of quantitative trait loci (QTLs), map-based gene cloning and marker assisted selection (MAS) for flax.

## Supporting information

S1 Fig SNP-based high-density genetic map of flax. SNP markers and their locations are shown on the right and left side, respectively. (PDF).
(PDF)

S2 Fig Haplotype maps of the genetic map. Each row represents a marker ranked in accordance with the map order. Each column represents a chromosome in each F_2_ individual. Green area represents R43 (female parent), blue area represents LH-89 (male parent), red area represents heterozygosity, and gray area represents missing data. (RAR).
(RAR)

S3 Fig Heat maps of the genetic map. Each cell represents the recombination rate of two markers. Yellow color indicates a lower recombination rate, which suggests a stronger linkage between markers, and purple a higher one, which suggests a weaker linkage between markers. (RAR).
(RAR)
