# Supplementary figures and images for: Construction of an SNP-based high-density linkage map for flax (Linum usitatissimum L.) using specific length amplified fragment sequencing (SLAF-seq) technology
Source: PLoS One. 2017 Dec 21;12(12):e0189785. doi: 10.1371/journal.pone.0189785 (PMC5739455; doi:10.1371/journal.pone.0189785)

# LG 1

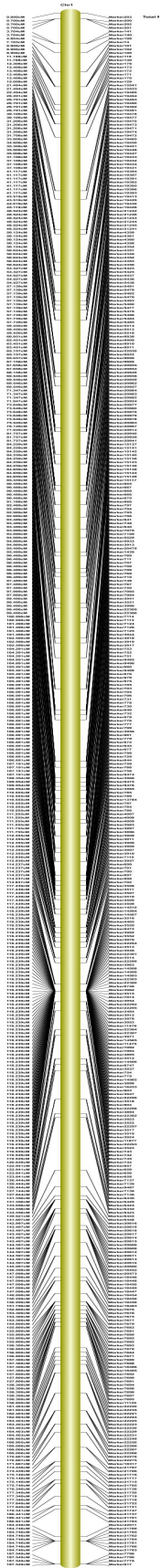

# LG 2

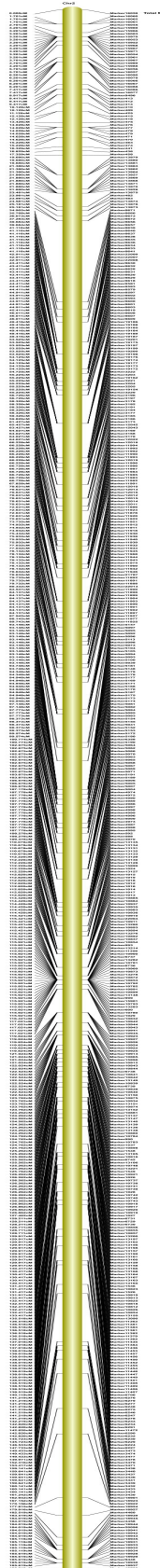

# LG 3

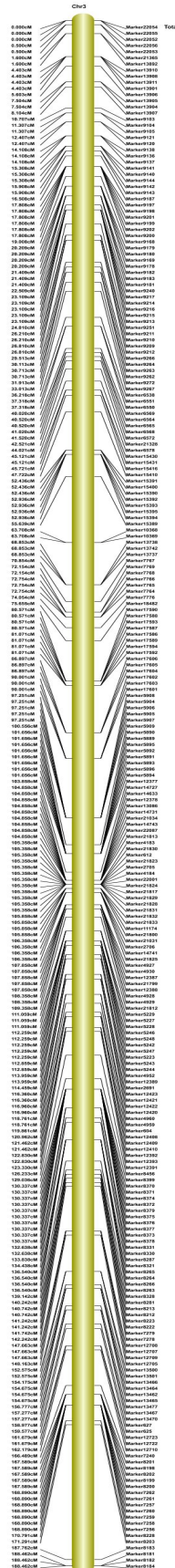

# LG 4

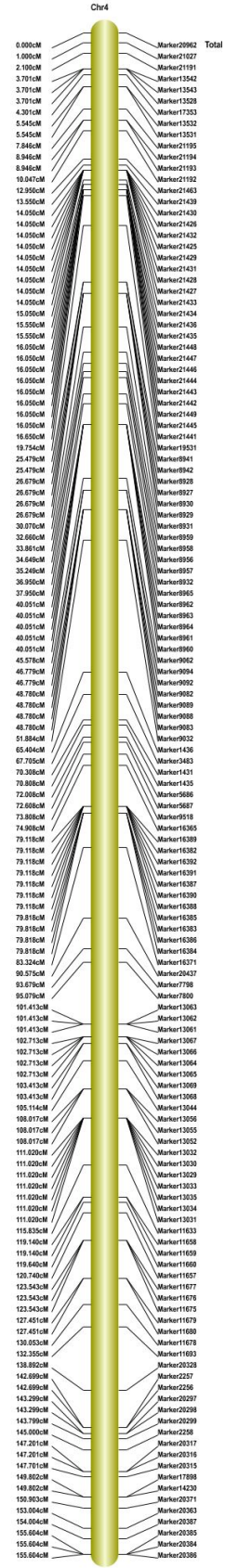

## LG 8

## LG 12

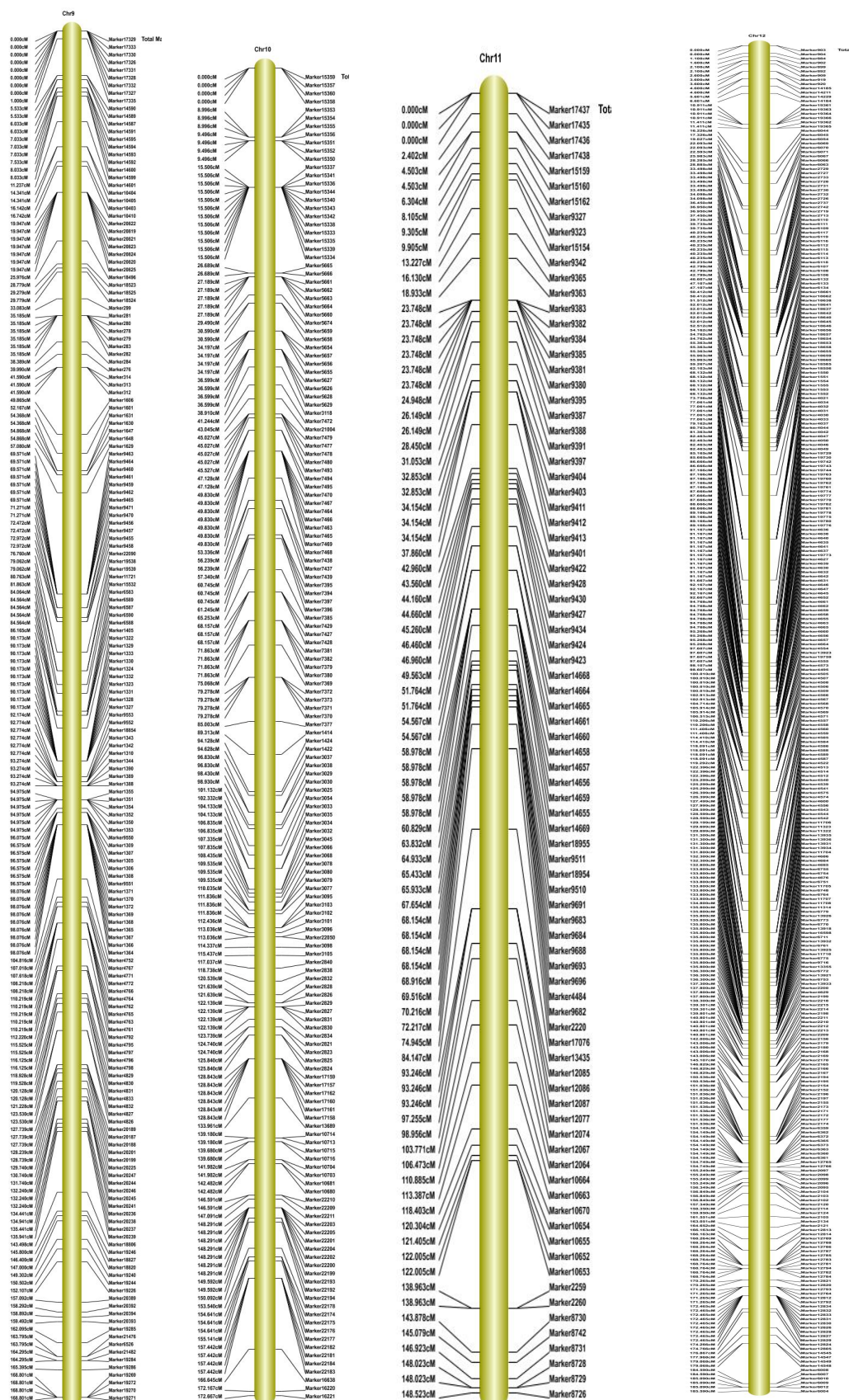

LG 13

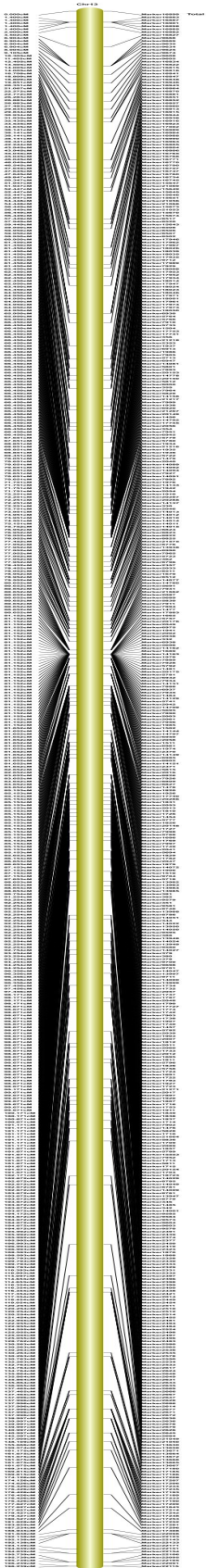

LG 14

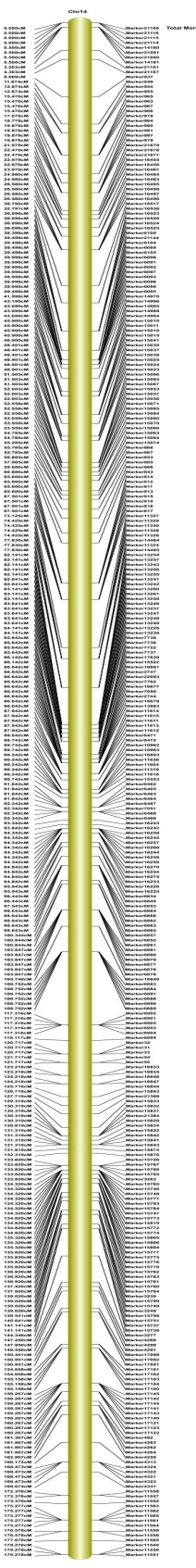

LG 15

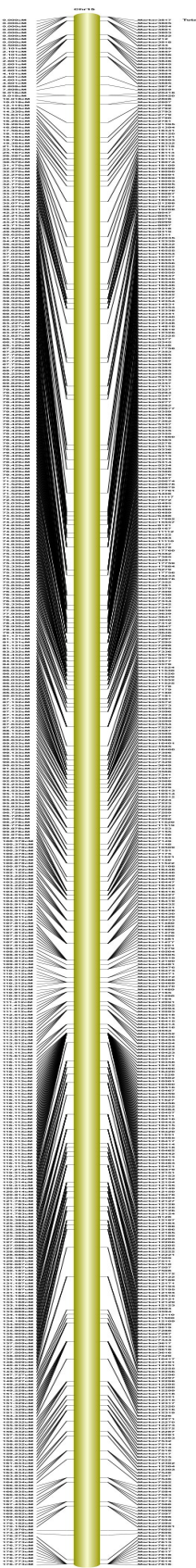

Supplement: S1 Fig — SNP markers and their locations are shown on the right and left side, respectively. (PDF). (PDF) [file pone.0189785.s001.pdf]
